# Supplementary material for: Removal Performance and Mechanism of Benzo(b)Fluorathene Using MnO2 Nanoflower/Graphene Oxide Composites
Source: Materials (Basel). 2021 Aug 6;14(16):4402. doi: 10.3390/ma14164402 (PMC8398222; doi:10.3390/ma14164402)
Supplement: Supplementary file 1 [file materials-14-04402-s001.zip › materials-1275266-supplementary.pdf]

materials

MDPI

Supporting Information

Removal Performance and Mechanism of Benzo[*b*]Fluorathene Using MnO_2 Nanoflower/Graphene Oxide Composites

Qingqing Cao [1], Siqi Lu [2], Wenjun Yin [1,2,*], Yan Kang [3], Naihao Yang [4], Yudong Hou [5] and Zizhang Guo [6,*]

1 School of Architecture and Urban Planning, Shandong Jianzhu University, Jinan 250014, China; caoqingqing18@sdjzu.edu.cn
2 College of Environmental Science and Engineering, Tongji University, Shanghai 200092, China; 1851284@tongji.edu.cn
3 College of Environment and Safety Engineering, Qingdao University of Science and Technology, Qingdao 266042, China; kangyan@qust.edu.cn
4 Jinan Engineering Consulting Institute, Jinan 250014, China; qdhfx.238@163.com
5 Majian International Architectural Design Consulting Co. LTD, Jinan 250014, China; hou.yu.dong@163.com
6 Shandong Key Laboratory of Water Pollution Control and Resource Reuse, School of Environmental Science and Engineering, Shandong University, Qingdao 266237, China
* Correspondence: yinwenjun1991@163.com (W.Y.); guozizhang@sdu.edu.cn (Z.G.)

Citation: Cao, Q.; Lu, S.; Yin, W.; Kang, Y.; Yang, N.; Hou, Y.; Guo, Z. Removal Performance and Mechanism of Benzo[*b*]Fluorathene Using MnO_2 Nanoflower/Graphene Oxide Composites. *Materials* **2021**, *14*, 4402. https://doi.org/10.3390/ma14164402

Academic Editor(s):Avelino Núñez-Delgado

Received:10 June 2021
Accepted:2 August 2021
Published: 6 August 2021

Publisher's Note: MDPI stays neutral with regard to jurisdictional claims in published maps and institutional affiliations.

Copyright: © 2021 by the authors. Licensee MDPI, Basel, Switzerland. This article is an open access article distributed under the terms and conditions of the Creative Commons Attribution (CC BY) license (http://creativecommons.org/licenses/by/4.0/).

Text S1:

Natural graphite powder (100 mesh, Alfa Aesar) was used to synthesize graphene oxide based on a modified Hummers' method. [1] Briefly, graphite power (0.5 g) and sodium nitrate (0.5 g) were first mixed with sulfuric acid (23 mL, 98%) at 0 °C followed by slow addition of 3 g potassium permanganate with constant stirring. Then, the mixture was kept at 35 °C for 1 h in a thermostat water bath. After that, the suspension was mixed with 40 mL deionized water and heated at 90 °C for 30 min. Subsequently, deionized water (100 mL) and hydroperoxide (5 mL, 30%) were added to terminate the reaction. Finally, the precipitate was separated by centrifugation at 5000 rpm for 15 min and washed with deionized water to neutral and dried at 60 ◦C for 12 h to get prepared graphene oxide (GO) for further use.

Text S2:

The morphology of as-prepared MnO_2 NF and MnO_2 NF/GO composite were observed by field emission scanning electron microscopy (JEOL 7800 FESEM/SEM, Freising, Germany) and microstructures were identified by high-resolution transmission electron microscopy (HRTEM, Zeiss Libra 200, Tokyo, Japan). [2-4] The HAADF (High-Angle Annular Dark Field)-STEM, elemental mapping, and EDX of composites were carried out using a Tecnai G2 F20 instrument at an accelerating voltage of 200 kV (Hillsboro, OR, USA. The crystallographic structure of as-synthesized powders was determined by X-ray diffraction (XRD, D/max-1200X, Cu Kα radiation (λ = 1.5406 Å) operating at 30 kV and 100 mA). The textural parameters including specific surface area, total pore volume, and N_2 adsorption/desorption isotherms were determined by Brunauer-Emmett-Teller (BET) (Quanta Chrome Corporation, Boynton Beach, FL, USA). The surface chemical properties (element composition and electronic valence) were investigated by X-ray photoelectron spectroscopy (XPS, Nico-let-460, Thermo Fisher, Zagreb, Croatia) and XPS data corresponding to C 1s, O 1s, and Mn 2p spectra were fitted using the software *Casa*XPS.

Table S1. Shimadzu GCMS-QP 2010 method parameters for BbF concentration determination.

GC	Inlet temperature		250 °C
	Sampling method		Unsplit stream sampling
	Sample size		2.0 μL
	Carrier gas		He, 1.5 mL/min
	Temperature programming	Initial column temperature	60 °C, held for 5 min
		Final column temperature	290 °C, held for 2 min
		Heating rate	10°C/min
	Retention time		35 min
MS	Ion source		EI
	Ion source temperature		225 °C
	Ionization Energy		70 eV
	Data acquisition and analysis		SIM (selected ion monitoring) mode
	Transfer line temperature		280 °C
	Electron multiplier voltage		Consistent with a tuning voltage

BbF-d12 standard (1000 mg/L, dissolved in CH2Cl2), Custom Semivolatile Mix of 2-Fluorobiphenyl, and P-Terphenyl-d14 (2000 mg/L, dissolved in acetone-hexane 1:1) used for the preparation of GC-MS samples were purchased from o2si smart solutions (made in the Shimadzu, USA).

References:

1. Hummers Jr, W.S.; Offeman, R.E., Preparation of graphitic oxide. *Journal of the american chemical society* **1958,** *80* (6), 1339-1339.
2. M. Xiang, O. Aguerre-Chariol, M. Morgeneyer, F. Philippe, Y. Liu, C. Bressot, Uncertainty assessment for the airborne nanoparticle collection efficiency of a TEM grid-equipped sampling system by Monte-Carlo calculation, Advanced Powder Technology, (2021).
3. M. Xiang, M. Morgeneyer, F. Philippe, M. Manokaran, C. Bressot, Comparative review of efficiency analysis for airborne solid submicrometer particle sampling by nuclepore filters, Chemical Engineering Research and Design, 164 (2020) 338-351.
4. M. Xiang, M. Morgeneyer, O. Aguerre-Chariol, F. Philippe, C. Bressot, Airborne nanoparticle collection efficiency of a TEM grid-equipped sampling system, Aerosol Science and Technology, (2021) 1-19.
